# Synergistic inhibitory effects of *Trifolium pratense *L. extract and doxorubicin on 4T1 tumor-bearing mice are mediated via targeting the Wnt/β-catenin pathway and reversal of epithelial-mesenchymal transition

**DOI:** 10.22038/ajp.2025.25940

**Published:** 2025

**Authors:** Saeed khazayel, Mohammad Hossein Faraji, Mohsen Akbaribazm, Mozafar Khazaei, Elham Niromand, Mohammad Rasool Khazaei

**Affiliations:** 1 *Fertility and Infertility Research Center, Health Technology Institute, Kermanshah University of Medical Sciences, Kermanshah, Iran*; 2 *Physiology Division, Department of Basic Science, School of Veterinary Medicine, Shiraz University, Shiraz, Iran*; 3 *Department of Anatomical Sciences, School of Medicine, Kermanshah University of Medical Sciences, Kermanshah, Iran*; 4 *Department of Basic Medical Sciences, Khoy University of Medical Sciences, Khoy, Iran*

**Keywords:** Trifolium pratense L., Doxorubicin, Epithelial-mesenchymal transition, Wnt signaling pathway, beta catenin

## Abstract

**Objective:**

Triple-negative breast cancer (TNBC) presents significant therapeutic challenges. This study investigates the combination effects of Trifolium pratense L. (red clover) and doxorubicin (DOX) on the Wnt/β-catenin signaling pathway, epithelial-mesenchymal transition (EMT) and apoptosis in 4T1 tumor-bearing BALB/c mice.

**Materials and Methods:**

Female BALB/c mice were divided into six (n=10) groups: control, DOX (5 mg/kg), and three treatment groups receiving 100, 200, or 400 mg/kg *T. pratense* extract alongside DOX, and a single dose of 400 mg/kg *T. pratense*. Tumor size was measured using Vernier calipers, and survival rates were analyzed through Kaplan-Meier curves. Tumors were removed to analyze histological examinations and gene expression of *Ccnd*, *Myc*, *Cdh1*, *Snai1*, *Sfrp2*, *Wif1*, *Kremen1*, and *ARHGAP17*. Immunohistochemical staining was performed to evaluate p53, Ki-67, β-catenin, Cdh1, and vimentin expression.

**Results:**

Co-treatment of *T. pratense* (400 mg/kg) with DOX (5 mg/kg) synergistically reduced cell proliferation and increased apoptosis by increasing p53 and decreasing Ki-67 expression in a dose-dependent manner. This co-treatment effectively inhibited the Wnt/β-catenin pathway by upregulating antagonists (*Wif1* and *Sfrp2*), modulating β-catenin accumulation, and reversing EMT through increased E-cadherin expression and decreased vimentin (protein level) and *Snai1 *(gene expression) levels.

**Conclusion:**

*T. pratense* extract shows potential as an adjuvant therapy against TNBC by targeting the Wnt/β-catenin pathway and reversing EMT while enhancing DOX efficacy. Further research is warranted to explore additional anticancer mechanisms of *T. pratense* extract.

## Introduction

Nowadays, breast cancer (BC) is considered the major cause of cancer death and the most common cancer in females worldwide (DeSantis et al. 2019). Estimations for 2023 indicate that BC held the top position among cancer diagnoses in the United States, constituting 31% of all cases (Siegel et al. 2023). Based on the literature, different signaling pathways are involved in breast cancer. Studies have revealed the role of overactivated Wnt signaling in initiating BC (Velloso et al. 2017; Yin et al. 2018). 

The involvement of the Wnt/β-catenin pathway in a variety of biological processes is well-established, and its aberrant activation has been implicated in the proliferation of tumor cells, particularly in the early stages of cancer development, as well as in the facilitation of metastasis. This pathway has emerged as a potential target for therapeutic interventions in BC (Khramtsov et al. 2010). The 4T1 cell line derived from the mammary gland of mice has been employed as a valuable tool for studying triple-negative breast cancer (TNBC). Through characterization, it has been observed that certain ligands and target genes associated with the Wnt/β-catenin pathway are significantly upregulated in 4T1 cells. This finding provides insights into the molecular mechanisms underlying TNBC and highlights the potential role of the Wnt/β-catenin pathway in this aggressive form of BC (Schrörs et al. 2020). Several studies have addressed the interaction between β-catenin and E-cadherin (Cdh1) within adherent junctions. The expression of *Cdh1* has been found to have an inverse relationship with invasion and tumor progression. A decrease in *Cdh1* expression levels is associated with an increased risk of epithelial-mesenchymal transition (EMT), leading to metastasis and invasion of breast carcinoma (Lombaerts et al. 2006). It is hypothesized that Cdh1 may function as a suppressor of the Wnt signaling pathway by sequestering cytoplasmic β-catenin (Borcherding et al. 2018; Mahmood et al. 2017; Tsai et al. 2013).

 There is an increasing fascination with the utilization of combination therapies and the exploration of plant-based drugs to decrease the adverse effects associated with existing treatments. It has been established that certain compounds, namely genistein, formononetin, and quercetin, possess inhibitory properties against human diseases by selectively targeting diverse cellular signaling pathways, such as the Wnt pathway (Fu et al. 2017; Khazayel et al. 2018; Kim et al. 2013; Srivastava and Srivastava 2019; Tsai et al. 2013).

Anthracyclines, such as doxorubicin (DOX), are a class of chemotherapeutic agents that exhibit anticancer properties. These drugs are derived from specific strains of *Streptomyces* bacteria, namely *S. caesius* and *S. peucetius*. DOX prompts the generation of free radicals and hinders the intercalation of topoisomerase II and DNA, and it has elevated the success rates of therapy from 40–50% to 60–80% (in conjunction with other medications such as curcumin, quercetin, and ocotillol) in the context of BC (Nadas and Sun 2006). The combination of these medications with other therapeutic agents can successfully trigger a range of anticancer pathways and enhance their safety characteristics by reducing the occurrence of side effects such as cardiotoxicity, thrombocytopenia, stomatitis, acute nausea, gastrointestinal disturbances, bone marrow aplasia, and vomiting (Lv et al. 2016).

*Trifolium pratense *L. (Red clover—*T. pratense*) has a high concentration of isoflavonoids (Saviranta et al. 2008). In the past, it was used as an ethnomedicine with a wide range of pharmacological actions and positive effects against various diseases (Kolodziejczyk‐Czepas 2016). Research has recently shown the anticancer effects of *T. pratense *and its contents of isoflavones against BC cells (Moon et al. 2008; Zhou et al. 2014) via increased expression of some apoptosis and autophagy-related genes (Khazaei et al. 2019) and anti-oxidant effects (Akbaribazm et al. 2021).

According to the positive effects of *T. pratense* extract against BC, the purpose of this study was to elucidate whether the combination of DOX with *T. pratense* can potentially suppress 4T1 breast cancer cells in BALB/c mice via affecting the Wnt signaling pathway.

## Materials and Methods

### Plant collection and extract preparation 

*T. pratense* seeds were provided by Karaj Seed and Plant Improvement Institute (Karaj, Iran; voucher No. KPC/ Kulubara-1274) and planted in the Kermanshah University of Medical Sciences research farm. To prepare the hydro-alcoholic extract, 300 g of flowers and leaves were powdered and dissolved in ethyl alcohol solution (70%). The solution was filtered by Whatman filter paper (No. 1, Millipore, USA). Also, a vacuum distillation unit (Heidolph Collegiate, LABOROTA 4000) at 55°C was used to compress the solution and obtain the extract at 4°C.

### Breast cancer induction

To induce BC, the 4T1 cell line (ATCC CRL-2539) was acquired from the National Cell Bank of Iran (Pasteur Institute, Iran). The cells were then cultured (>85% viability) in RPMI 1640 medium under standard cell culture conditions including 5% CO_2_ (Memmert CO2 incubator, INCO153med, Germany), 1% penicillin-streptomycin, and 10% fetal bovine serum (FBS) at 37°C (Sigma, USA). A certain number of cells (1×10^6^) were counted, diluted with phosphate buffered saline (PBS), and inoculated subcutaneously into the right mammary fat pad. 

### Study groups and treatment

A total of 60 female 7-week-old BALB/c mice (n= 10 per group) were purchased from the Pastor Institute (Tehran, Iran). The mice were housed in normal physiological conditions (12 hr of light followed by 12 hr of darkness, 50±10% humidity, and 24±2°C temperature) and anesthetized with ketamine 10% (15 mg/kg/ intraperitoneal (i.p.)) and xylazine 2% (80 mg/kg/ i.p.) on day 45 of the study according to standard laboratory animal protocols and recommendation of the Ethics Research Committee of the Kermanshah University of Medical Sciences (Ethics code: IR.KUMS.REC.1398.359), the ARRIVE guidelines 2.0 and Animal Ethics Committee (NIH Publication 80–23, 1996). The mice were divided into the following six groups: 

The control group (C) received 0.5 ml of distilled water by gavage. 

Doxorubicin group (DOX) received 5 mg/kg/week of DOX intravenous as a positive control. 

**Figure 1:**
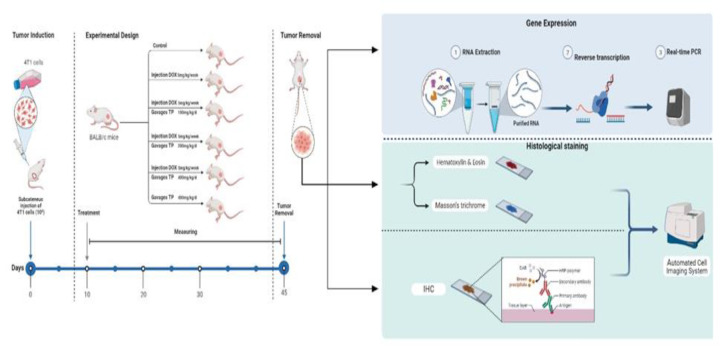
Experimental design and research timeline. TP: Trifolium pratense, DOX: doxorubicin, IHC: Immunohistochemistry.

The three synergism treatment groups received a single dosage of 5 mg/kg of DOX (D) in combined with hydroalcoholic extracts of *T. pratense* (t) at different concentrations of 100 (D+t100), 200 (D+t200), and 400 (D+t400) mg/kg by gavage for the assay of synergistic effects. Group six received only 400 mg/kg of *T. pratense* extract (t400). The extract* of T. pratense *treatment was administered every 24 hr at specified times of the day for 35 consecutive days (Figure 1).

### Measurement of tumor size and survival assay

After treatment, the survival rate, tumor size (assayed with Vernier callipers), and body weight were examined on specific days. To calculate tumor volume, the length and width of each tumor were measured with the following formula:

 Volume (cm^3^) =[(width)^2^×length]/2. The Kaplan-Meier curve was used to evaluate the survival rate. 

### Immunohistochemically assay

To perform immunohistochemical (IHC) staining of p53, Ki-67, β-catenin, Cdh1, and vimentin proteins, the tumor tissues from mice were fixed in a solution containing 10% formalin and then, embedded in paraffin blocks. Subsequently, sections with a thickness of 3-4 μm were prepared from the paraffin blocks of breast tumors using an RM2125 RTS instrument manufactured by Leica Inc., United States. The prepared slides were then subjected to an overnight incubation at 4°C with specific antibodies targeting p53 (Dako, cat.M7001), Ki-67 (Dako, cat.M7240), β-catenin (Santa Cruz Biotechnology, inc. cat.sc-7963), Cdh1 (Dako, cat.M3612), and vimentin (Dako, cat.M0725). Following this, the sections underwent deparaffinization using xylene and rehydration using graded concentrations of alcohol. To block endogenous peroxidase activity, the sections were exposed to a 3% H_2_O_2_ solution and subsequently blocked with 5% bovine serum albumin (BSA). For detection, the sections were incubated with a biotinylated rabbit anti-mouse IgG antibody (Biolegend, San Diego, USA), followed by streptavidin-horseradish peroxidase. The visualization of the target proteins was achieved using 1, 3-diaminobenzidine (DAB) tetrahydrochloride, and the sections were counterstained with hematoxylin. To analyze the stained slides, cytation 5 Cell Imaging Multi-Mode Reader (BioTek) was utilized. The number of positive cells was quantified in 10 fields of view for each slide and is reported as the mean percentage of positive cells ±SEM (standard error of the mean).

### RNA extraction

GeneMATRIX Universal RNA Purification Kit (EURx Sp. z o.o, Cat.No. E3598-02, Poland) was used for total RNA isolation. First, 20 mg of tumor tissues was grinded into a fine powder. Next, the DNA was removed using a homogenization spin-column and the RNA was extracted using an RNA binding spin-column.

### cDNAs synthesis

Following the evaluation of total RNAs integrity, 1 μg of the total RNA was transformed to complementary DNA (cDNA) by reverse transcription PrimeScript™ RT reagent Kit (Perfect Real Time) (Takara Bio Inc. Japan, Cat. No. RR037A). To synthesize cDNA, the following reaction mixture was prepared on ice, 2 μl PrimeScript buffer, 0.5 μl of each primer (Random hexamers (100 μM) and Oligo dT (50 μM)), 0.5 μl of PrimeScript RT Enzyme Mix, and 1 μg of total RNA which finally adjusted total valume to 10 μl with RNase Free distilled water (dH_2_O). Then, the mixture was incubated at 37°C for 15 min, and 85°C for 5 sec.

### Real-time PCR

Real-time PCR was performed using TB Green® Premix Ex Taq™ (Takara Bio Inc. Japan, cat.no. RR420A). Based on the Applied Biosystem Real-Time PCR System (Step one), we prepared a real-time mixture with 10 μl of TB Green Premix Ex Taq, 0.4 μl of each primer, 0.4 μl ROX Reference Dye, and 1 μl templet and adjusted it to 20 μl volume with sterile purified water. *GPDH* was applied as a housekeeping gene, and data was calculated via the 2^-ΔΔCT^ formula. The primers for some genes were designed using AlleleID software (V. 7.5, Premier Biosoft, USA) and NCBI primer-BLAST (https://www.ncbi.nlm.nih.gov/tools/primer-blast/). Table 1 shows the sequence of the primers used in this study. 

**Table 1:** Primer Sequences utilized for RT-PCR amplification.

**Gene**	**Accession Number**	**Annealing temperature, °** ***C***	**Size (bp)**	**Primer sequence**	**Reference**
*Gapdh*	NM_008084	60	223	F: 5ˊ-AACTTTGGCATTGTGGAAGG-3ˊ R: 5ˊ-ACACATTGGGGGTAGGAACA-3ˊ	(Yoshiga et al. 2008)
*Arhgap17*	NM_001122640	60	126	F: 5ˊ-ACGGCATAGCAGAGGTGGAG-3ˊ R: 5ˊ-AGTTGGTTCCTGAAGACTTGTG-3ˊ	This study
*Cdh1*	NM_009864	60	142	F: 5ˊ-GATCCTGCTGCTCCTACTGT-3ˊ R: 5ˊ-GCTCAAATCAAAGTCCTGGTCT-3ˊ	(Chala et al. 2017)
*Ccnd1*	NM_007631	60	183	F: 5ˊ-GCGTACCCTGACACCAATCTC-3ˊ R: 5ˊ- CTCCTCTTCGCACTTCTGCTC-3ˊ	(Feng et al. 2009)
*Myc*	NM_010849	60	176	F: 5ˊ-CGCCTACATCCTGTCCATT-3ˊ R: 5ˊ-AACCGTTCTCCTTACTCTCAC-3ˊ	This study
*Kremen1*	NM_032396	60	100	F: 5ˊ-CGGGCACCAGTAAAACCTCT-3ˊ R: 5ˊ-GCATAGCCTGACTCCATCCC-3ˊ	This study
*Snai1*	NM_011427	60	133	F: 5ˊ-CACACGCTGCCTTGTGTCT-3ˊ R: 5ˊ-GGTCAGCAAAAGCACGGTT-3ˊ	(Lee et al. 2006)
*Sfrp2*	NM_009144	60	182	F: 5ˊ-CAAGAATGAGGACGACAACG-3ˊ R: 5ˊ-ACAGCACGGATTTCTTCAGG-3ˊ	This study
*Wif1*	NM_011915	60	104	F: 5ˊ-CGATGTATGAACGGTGGTCTG-3ˊ R: 5ˊ-AAGCAGGTGGTTGAGCAGTT-3ˊ	This study

### Histopathological Staining

#### Hematoxylin and Eosin (H&E) Staining

Tissue sections were deparaffinized, rehydrated through graded alcohols, and stained with hematoxylin to highlight cell nuclei. Following hematoxylin staining, sections were rinsed, differentiated, and counterstained with eosin to provide contrast for cytoplasmic structures. The stained sections were then dehydrated, cleared, and mounted for histological examination.

### Masson's Trichrome Staining

Sections were prepared as described above and stained using Masson's trichrome protocol to visualize collagenous connective tissue fibers. Sections were first stained in an acidic dye solution to color the cytoplasm and muscle fibers. Collagen fibers were then stained with an aniline blue or green dye, highlighting connective tissue elements.

### Statistical analysis

The data's normal distribution was evaluated through the Shapiro-Wilk and Levene tests for homogeneity of variances (p>0.05). Results are presented as mean±SEM, with all trials performed in triplicate. To compare mean differences between the groups, a One-Way ANOVA test was conducted, followed by the Dunnett t *post hoc* test (2-sided). Data analysis was carried out using SPSS software (V. 21, SPSS Inc., Chicago, IL), with statistical significance set at p<0.05. Survival rate analysis was performed using the Kaplan-Meier test in GraphPad Prism software (GraphPad Software, Boston, Massachusetts USA, www.graphpad.com version.8.0.2).

## Results

### Effects of T. pratense on survival rate, body weight, and tumor volume

**Figure 2:**
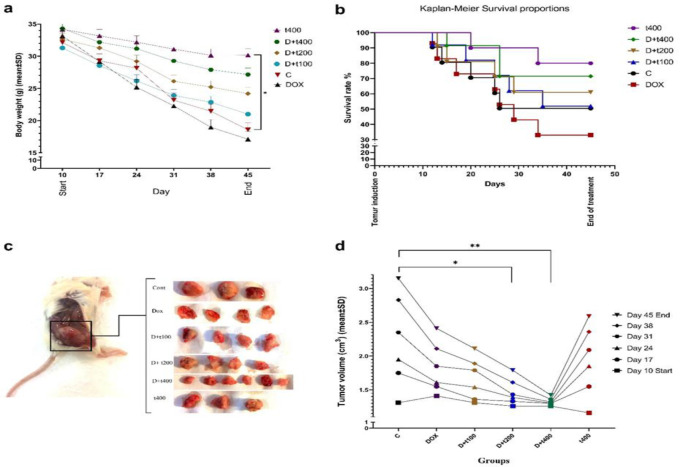
The effect of Trifolium pratense on a) Body weight of treatment groups b) survival rate (Kaplan–Meier curve), c) 4T1-tumor bearing mice and tumor samples, d) The volume of tumors (cm3) on day 10, 17, 24, 31, 38 and 45. . Data are reported as mean±SEM of three independent assays. Signiﬁcant alterations are expressed relative to control and marked with asterisks. *p<0.05 or **p<0.01.

Following the induction of tumors in mice, the weights of the animals were assessed on specific days (days 10, 17, 24, 31, 38, and 45). The t400 and D+t400 groups exhibited higher weight gains compared to the other groups. Notably, a statistically significant disparity in weight gain was noted in the t400 group (p=0.0467) when compared to the control group. Furthermore, there was a significant variance between the t400 (p=0.0148) and DOX groups (Figure 2a). After tumor induction, a declining pattern in tumor volume was observed in D+t200 (p=0.0159) and D+t400 (p=0.0035) in contrast to the control group (Figure 2c and 2d). Figure 2d illustrates the survival curve, indicating an upward trend in the survival rates of mice in the t400 and D+t400 groups, with survival rates of 80% and 70%, respectively. The Log-rank test, commonly employed for comparing survival analysis across multiple groups, revealed a significant difference in survival distribution among the groups (p=0.026).

### Histopathological findings

**Figure 3:**
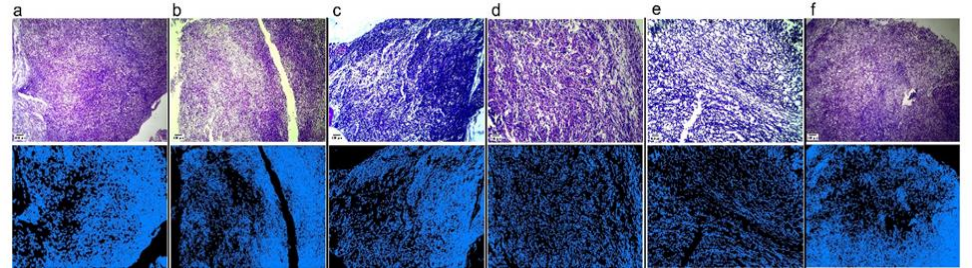
Evaluation of histopathological changes by hematoxylin and eosin staining. The top row photos are the hematoxylin and the bottom row was analyzed by Avizo^™^ Software. a) control, b) Dox, c) D+t100, d) D+t200, e) D+t400, and f) t400. Scale bar = 400 μm.

**Figure 4:**
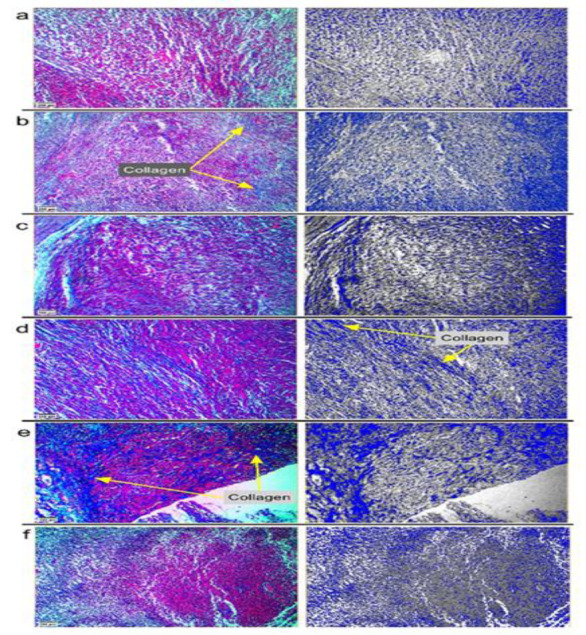
Masson's trichrome staining was conducted to highlight collagenous connective tissue fibers. a) control, b) Dox, c) D+t100, d) D+t200, e) D+t400 and f) t400. Synthesis of intercellular collagen fibers was increased (Yellow arrow) in the D+t400 group. To better observe histological changes for the synthesis of collagen fibers, the images were analyzed by Thermo Scientific™ Avizo™ Software. The highlighted blue area is indicative of the presence of collagen. Scale bar = 200 μm.

H&E staining revealed that the extracellular space was increased in the Dox+t400 treated group, and cell density was decreased, indicating the replacement of 4T1 cells with parenchymal tissue. To better observe histological changes among the groups, the image was analyzed by Thermo Scientific™ Avizo™ Software (Avizo 9.0.1 (c) 1995-2015 Zuse Institute Berlin (ZIB)). In this analysis, the area object surface was identified by determining the interactive thresholding (Binarization), separate objects, and label analysis. Finally, objects were filtered to better understand the histological changes (Figure 3). The Masson's trichrome staining was used for the assessment of collagenous connective tissue fibers in histological sections. Connective tissue and collagen fiber synthesis showed an increase in the Dox-t400 groups (Figure 4).

### T. pratense with DOX downregulated the Wnt/β-catenin pathway and reversed EMT

**Figure 5:**
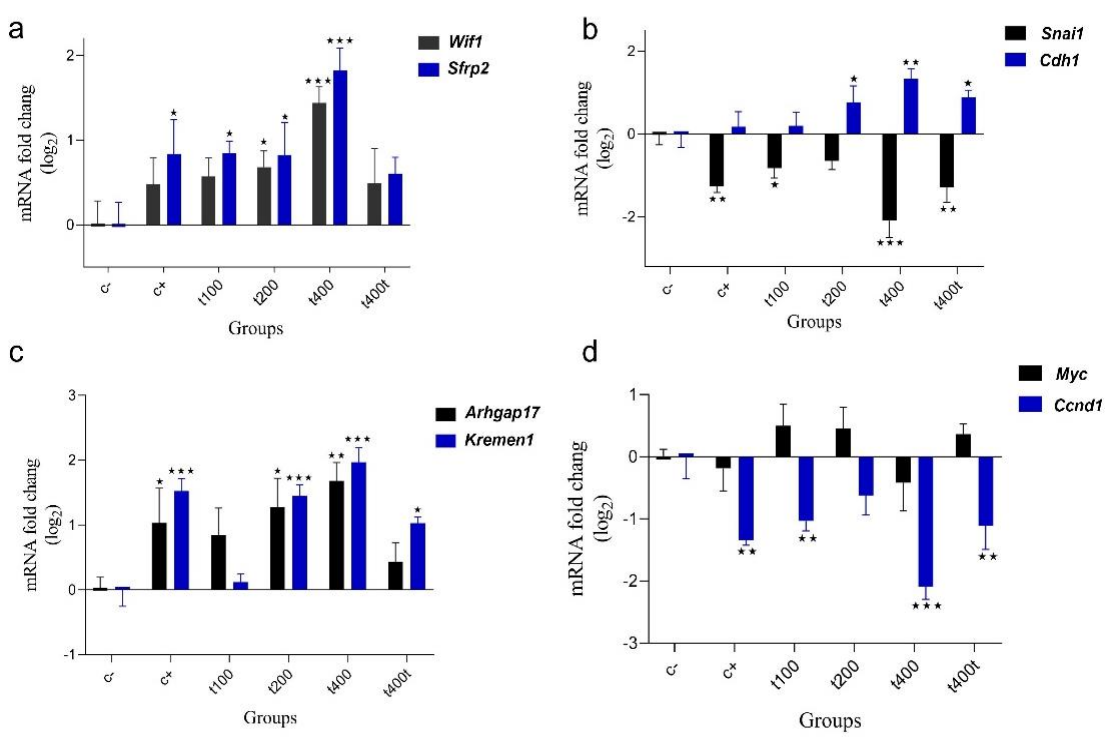
The effect of Trifolium pratense on gene expression of a) Wif1, and Sfrp2, b) Snai1, and Cdh1, c) Arhgap17, and Kreman1, d) Myc, and Ccnd1. Data are reported as mean±SEM of three independent experiments. Signiﬁcant alterations are expressed relative to control and marked with asterisks. *p<0.05, or **p<0.01, or ***p<0.001.

To assess the gene expression of the Wnt pathway and EMT, the mRNA levels of *Cdh1*, *Kremen1*, *Sfrp2*, Rho GTPase Activating Protein17 (*ARHGAP17*), Wnt inhibitory factor 1 (*Wif1*), *Snai1*, *Myc*, and *Ccnd1* were quantified. In Figure 5, mRNA levels of *Cdh1* (fold change log2 =1.33333; p<0.001), *Kremen1* (fold change log2 = 1.97000; p<0.001), *Sfrp2* (fold change log2 =1.81333; p<0.001), *Arhgap17* (fold change log2 = 1.64700; p=0.0012), and *Wif1* (fold change log2 = 1.42267; p< 0.001) were elevated in the D+t400 group compared to the control. Conversely, the mRNA levels of *Snai1* (fold change log2 = -2.07333; p<0.001), *Myc* (fold change log2 = -0/42967; not significant), and *Ccnd1* (fold change log2 = -2.08800; p<0.001) were reduced in the D+t400 group compared to the control group.

**Figure 6:**
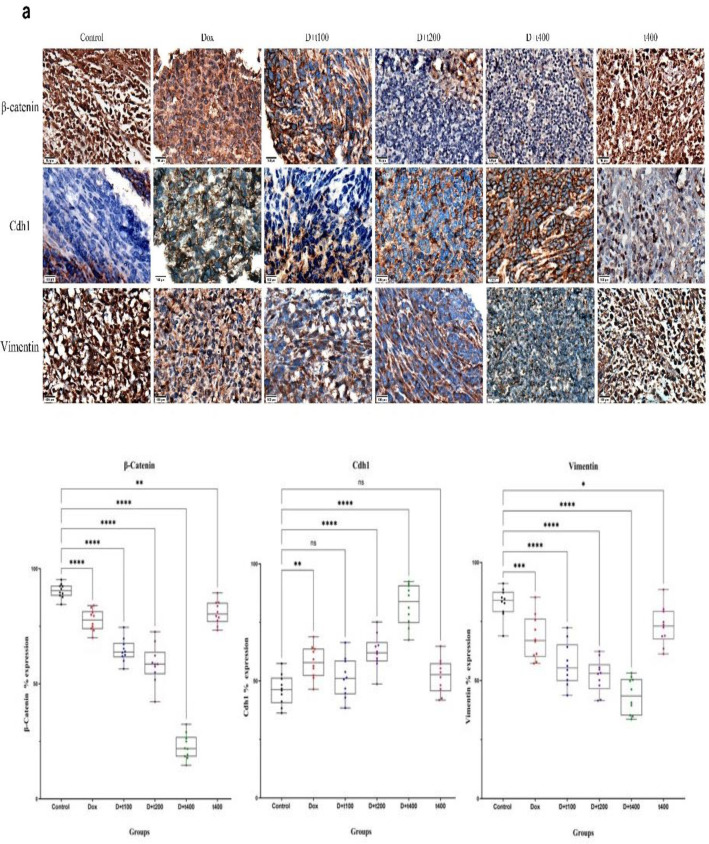
a) The effect of Trifolium pratense on β-catenin, Cdh1 (E-cadherin), and vimentin protein expression. immunohistochemical presents 3 independent blots per analysis. Positive and negative cells were counted in each field (10 fields/tumor) for b) β-catenin, c) Cdh1, and d) vimentin. data are reported as Mean±SEM. Scale bar = 100 μm. Signiﬁcant alterations are expressed relative to control and marked with asterisks. ns = not significant, *p<0.05, or **p<0.01, or ***p<0.001, ****p<0.0001.

Tumors were also stained using immunohistochemical (IHC) for β-catenin, Cdh1, and vimentin proteins to evaluate the co-treatment effect of *T. pratense *extraction and DOX on the Wnt pathway and EMT. In the control group, the β-catenin protein was found to accumulate in the nucleus. However, in the D+t200 and D+t400 groups, there was a significant reduction in the accumulation of β-catenin (p<0.001) compared to the control. The expression of Cdh1and vimentin proteins showed an inverse relationship. The gene expression of *Cdh1* increased in the D+t200 and D+t400 groups (p=0.0036), while vimentin (protein expression) significantly decreased in these groups (p<0.001) compared to the control (Figure 6). 

### Co-administration of T. pratense with DOX inhibited proliferation and induced apoptosis rate

**Figure 7:**
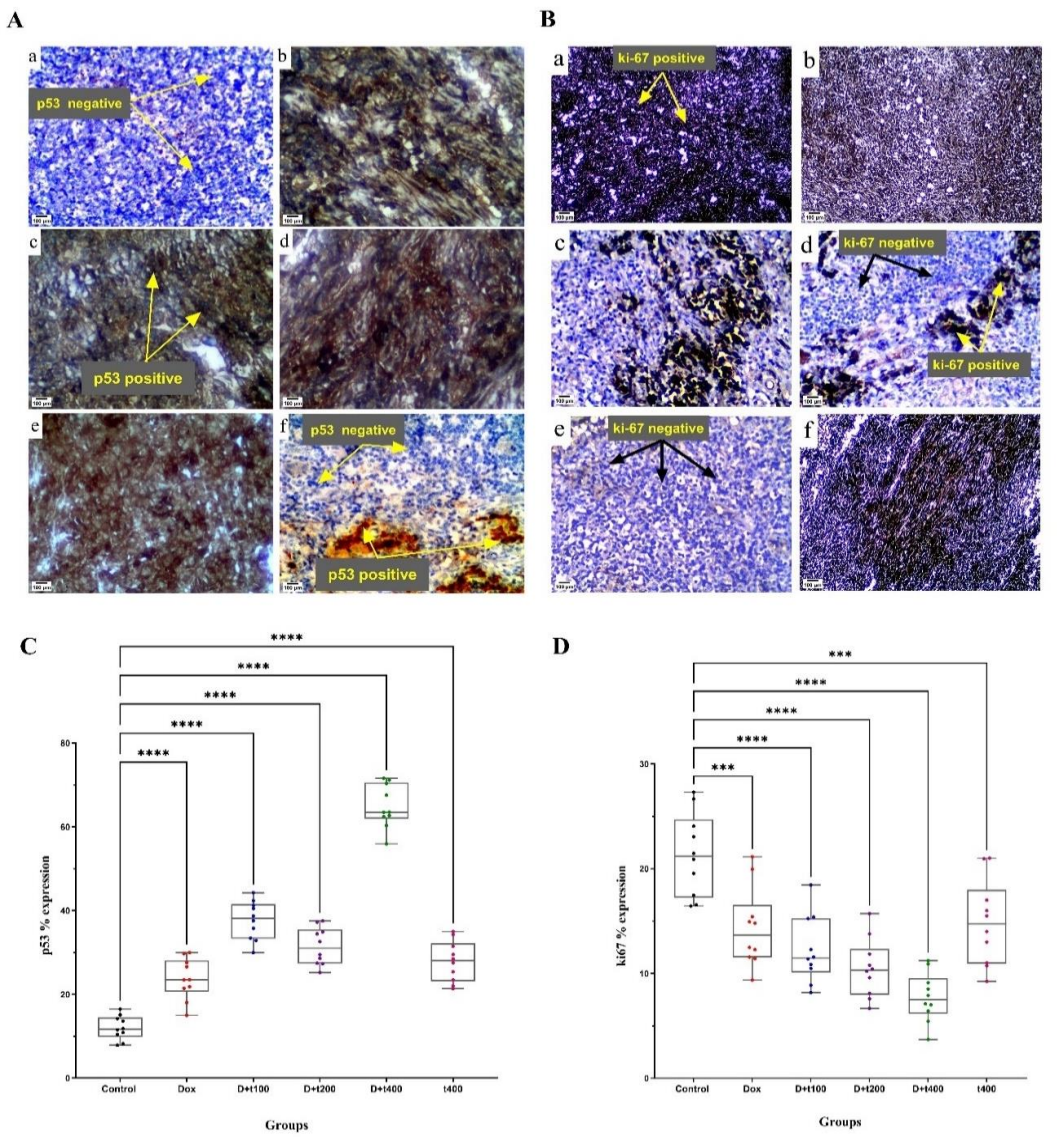
The effect of Trifolium pratense on A) p53 and B) Ki-67 protein expression. Immunohistochemical presents 3 independent blots per analysis. a) control, b) DOX, c) D+t100, d) D+t200, e) D+t400, and f). Positive and negative cells were counted in each field (10 fields/tumor) for C) p53 and D) Ki-67. Data are reported as Mean±SEM. Scale bar = 100 μm. Signiﬁcant alterations are expressed relative to control and marked with asterisks. ***p<0.001, ****p<0.0001.

The impact of co-administration of *T. pratense* with DOX on apoptosis and proliferation rates was investigated by staining tumors with immunohistochemical for p53 and ki-67 protein levels. Figure 7 demonstrates that the protein expression of p53 was significantly elevated in the D+t200 (p=0.003) and D+t400 (p<0.001) groups compared to the control group. Meanwhile, the protein expression of ki-67 was reduced in the treatment groups of D+t200 (p=0.0001) and D+t400 (p<0.001) compared to the control group.

## Discussion

The results provided strong evidence that the combination of *T. pratense* extract and DOX effectively suppressed the growth of 4T1 cells in tumor-bearing BALB/c mice. In the group that received both DOX and *T. pratense* (400 mg/kg), the tumor size decreased in a dose-dependent manner, and the mice showed improved survival rates and increased body weight compared to the control group. These findings contradicted the results of our previous study (Akbaribazm et al. 2020b).

Despite its value as an anticancer medication in BC treatment, the use of DOX is restricted due to its toxic effects, including oxidative stress and inflammation (Pugazhendhi et al. 2018). Consequently, it is advisable to administer complementary compounds alongside DOX to mitigate these toxic effects. The levels of β-catenin protein have been linked to DOX chemoresistance (Merikhian et al. 2021). Therefore, by reducing β-catenin in TNBC cell lines, resistance to DOX chemotherapy is diminished, resulting in the formation of smaller tumors in xenograft mice models (Xu et al. 2015).

Isoflavones, one of the most important bioactive substances, are abundant in *Trifolium pratense* (red clover). Acidified aqueous methanol is used to extract these isoflavones, followed by an analysis using ultra-performance liquid chromatography. The study revealed the presence of different types of isoflavones in *T. pratense* extract, namely formononetin (51%), biochanin A (40%), daidzein (2%), and genistein (7%) (LEMEŽIENĖ et al. 2015). It was discovered that these isoflavones had specific antioxidant qualities (Akbaribazm et al. 2020a). Furthermore, several studies have indicated that isoflavone compounds can inhibit the Wnt/β-catenin signaling pathway and EMT, as suggested by previous research (Amawi et al. 2017; Kim et al. 2013; Sarkar et al. 2010). Quercetin suppresses the Wnt/β-catenin signaling pathway through the upregulation of Wnt pathway inhibitors (*DKK1*, *2,* and *3*) (Kim et al. 2013). A formononetin-coumarin hybrid compound, derived from formononetin, demonstrated anti-proliferative, tumor growth inhibitory, and anti-migratory properties against SGC7901 gastric cancer cells by modulating the Wnt/β-catenin and Akt/mTOR pathways (Yao et al. 2019).

Formononetin-dithiocarbamate derivatives, another variant of formononetin, were found to have the ability to suppress Wnt/β-catenin signaling. This suppression resulted in the inhibition of growth and migration of PC-3 cells. The mechanism behind this inhibition involved a decrease in the expression of β-catenin and TCF-4 proteins, as well as an increase in the expression of Axin scaffold protein (Fu et al. 2017). In line with our results, Lin et al. reported similar effects of formononetin-derivatives in the treatment of MDA-MB-231 malignant tumors (Lin et al. 2017). In the context of colon cancer cell lines, Biochanin-A (Bio-A) is recommended as an adjuvant with 5-fluorouracil (5FU) to reduce drug toxicity and tumor resistance. This combination led to an increase in the ratio of phosphorylated β-catenin (p-S45) to total β-catenin. The mechanism behind this increase involved the downregulation of Akt-phosphorylation and the augmentation of GSK3β (active form) (Mahmood et al. 2017).

**Figure 8:**
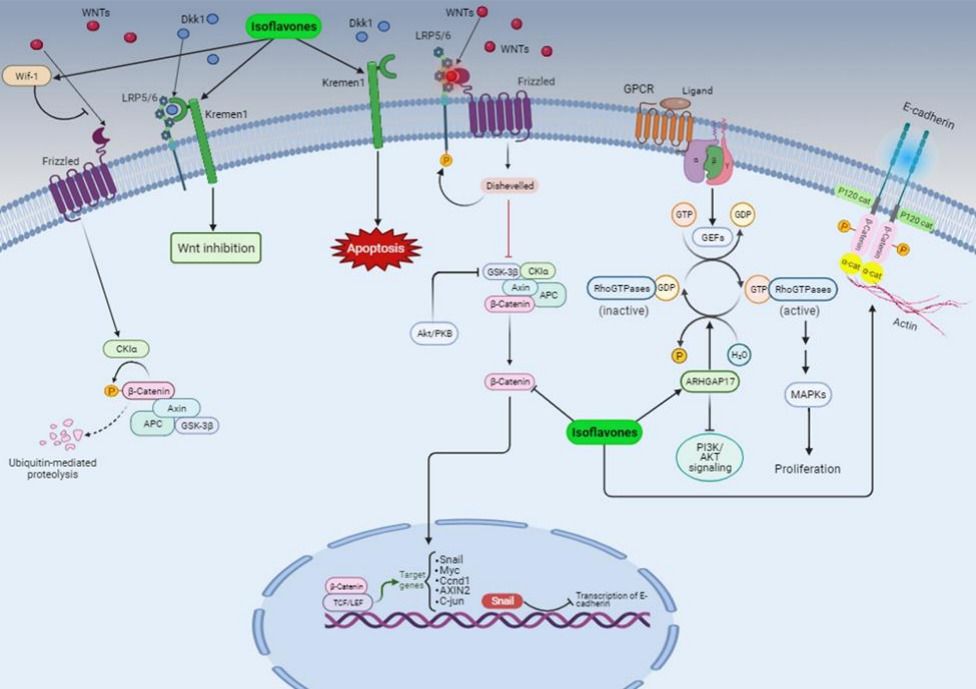
*The outcomes of this investigation are visually presented schematically, illustrating the influence of isoflavones extracted from *Trifolium pretense*. The isoflavonoids were observed to exert a downregulatory effect on the Wnt/β-catenin pathway, leading to the reversal of epithelial-mesenchymal transition (EMT). This reversal was achieved by inhibiting the binding of WNTs to their respective receptors, while simultaneously enhancing the expression of the Kerman receptor, Arhgap17, and E-cadherin. Additionally, the isoflavones were found to decrease the quantity of β-catenin protein.*

Kreman1 functions as a transmembrane receptor that interacts with the secretory protein Dickkopf-1 (Dkk1), which is recognized as an extracellular Wnt-antagonist (Causeret et al. 2016; Kim et al. 2013). This receptor plays a dual role in cellular processes. Firstly, Osada et al. showed that Kreman1 facilitates the binding of Dkk1 to the inactive form of LRP5/6, thereby forming a complex with Dkk1/LRP5/6 at the cell membrane (Osada et al. 2006). Secondly, Sumia et al. determined that Kreman1 can induce caspase-3-mediated cell death, even in the presence of Dkk1 (Sumia et al. 2019). These findings, along with our previous research indicating elevated caspase-3 expression (Akbaribazm et al. 2020b), are consistent with the results of the current study, which demonstrate an increase in Kreman1 and p53 levels, as well as a decrease in Ki-67 expression. Therefore, Kremen1 not only inhibits Wnt/β-catenin signaling but also triggers apoptosis (Figure 8).

Based on our findings, *T. pratense* extract at doses of 100, 200, and 400 mg/kg can up-regulate the expression of secreted Frizzled-related protein 2 (*SFRP2*) and *Wif1*, both acting as antagonists to Wnt signaling. Zhang et al. revealed that treating colon cancer cells (DLD-1) with 75 μM/L of genistein resulted in a reduction of promoter methylation of the *SFRP2* gene by up to 50%, a result comparable to that of 5-aza-cytidine, a DNA methyltransferase inhibitor. The up-regulation of *SFRP2* gene expression led to the inhibition of nuclear β-catenin protein and the attenuation of aberrant WNT signaling (Zhang et al. 2011). Furthermore, Zhu et al. demonstrated that genistein at 60 μM can trigger *Wif1* demethylation and restore its expression in HT29 colon cancer cells. Following genistein treatment, both mRNA and protein levels of* Cdh1* were elevated. Conversely, the expression of *β-catenin*, *Myc*, and *Ccnd1* was notably reduced, leading to the down-regulation of Wnt/β-catenin signaling. Additionally, genistein can revive *Wif1* expression in HT29 colon cancer cell lines by modifying *Wif1* methylation (Zhu et al. 2018).

Wnt/β-catenin signaling is synergistically related to EMT (Tsai et al. 2013). β-catenin not only acts as an essential regulatory protein in Wnt signaling transmission but also plays a vital role in cadherin-mediated cell adhesion and connected membrane‐associated Cdh1 to the cellular actin cytoskeleton (Amawi et al. 2017) (Figure 8). When β-catenin is detached from the cytoplasmic tail of Cdh1, it results in an increase of free β-catenin in the cytoplasm, leading to the migration of epithelial cells (Tsai et al. 2013). Epithelial cells typically have a high expression of Cdh1, while mesenchymal cells show increased expression of proteins like N-cadherin and vimentin (Francou and Anderson 2020). In MDA-MB-231 and 4T1 cells, metastasis is prevented by reversing EMT through the enhancement of p-β-catenin, GSK-3β, Cdh1, and decreased levels of vimentin (Chen et al. 2019). Similar to our results, Zhang et al. have noted that the effect of genistein on human cancer cell lines leads to reduced EMT levels by diminished levels of vimentin and increased status of E-cadherin in a dose-dependent manner (Zhang et al. 2008). Additionally, Liu et al. showed that formononetin could enhance the sensitivity of glioma cells to DOX by inhibiting histone deacetylase 5 and thereby reversing EMT (Liu et al. 2015). Consequently, the combination therapy of DOX and isoflavones, when used as an adjuvant agent, has the potential to enhance the therapeutic efficacy of DOX.

The process of EMT is subject to regulation by a variety of transcription factors, among which the zinc-finger factor *Snai1* stands out as a major inducer of EMT in BC. By binding to three E-box domains in the proximal E-cadherin promoter, the Snai1 inhibits transcription of *Cdh1*, This inhibition ultimately leads to an increased release of β-catenin (Yook et al. 2006). Studies have demonstrated that NF-κB can enhance the expression of Snai1, while genistein inhibits the EMT process by targeting NF-κB and reducing GSK-3β activities (Lin and Li 2015). On the other hand, biochanin A and formononetin have been found to suppress the NF-κB and Akt pathways (Cho et al. 2017; Ong et al. 2019). Our experimental results revealed that the administration of *T. pratense* (400 mg/kg) in combination with DOX effectively reversed EMT in 4T1 tumor cells, indicating that isoflavonoids may modulate Snai1 expression by inhibiting NF-κB.

In recent times, the role of Rho GTPase Activating Protein17 (*ARHGAP17*) as a tumor suppressor and negative regulator for *Cdc42* and *Rac1* has been the subject of investigation by researchers (Guo et al. 2019; Pan et al. 2018). A study conducted by Pan et al. has provided evidence that *ARHGAP17* is downregulated in 85 colon cancer patients and exhibits a negative correlation with the levels of *β-catenin*, *Myc*, and *MMP7* mRNA (Pan et al. 2018). Our findings confirmed that the mRNA level of *ARHGAP17* was increased in the group treated with DOX and T*. pratense* 400 mg/kg, suggesting that *ARHGAP17* may serve as a potential tumor suppressor against TNBC xenografts.

In summary, the results of the present study showed that 400 mg/kg of *T. pratense* extract (as an adjuvant agent) has a potential anticancer activity that could enhance the therapeutic efficacy of DOX in 4T1 tumor-bearing BALB/c mice via downregulation of Wnt/β-catenin pathway and EMT. Further experiments seem necessary to determine other possible mechanisms for the anticancer effects of *T. pratens*e extract. However, enhanced comprehension of the fundamental processes implicated in the potential anticancer activity of *T. pratense* extract, and the co-treatment of *T. pratense* extract with other standard chemotherapeutic drugs against BC is needed to clarification of anti-cancer mechanisms for future research. Furthermore, lack of awareness of the bioavailability of *T. pratense* extract ingredients and failure to measure important proteins involved in the Wnt/β-catenin pathway and EMT by western blot is regarded as the limitations of the present study.
